# Pathological response and survival outcomes after neoadjuvant chemotherapy with radical cystectomy in octogenarians for muscle-invasive bladder cancer: an observational database study

**DOI:** 10.1186/s12894-024-01548-y

**Published:** 2024-07-24

**Authors:** Arjun Pon Avudaiappan, Pushan Prabhakar, Mayer Simcha Sandman, Muni Rubens, Rohan Garje, Ahmed Eldefrawy, Murugesan Manoharan

**Affiliations:** 1 Department of Urologic Oncology Surgery, Miami Cancer Institute, 8900 N Kendall Drive, Miami, FL 33176 USA; 2 Herbert Wertheim College of Medicine, Florida International University, 11200 SW 8th St, Miami, FL 33199 USA

**Keywords:** Muscle-invasive bladder cancer, Octogenarians, Radical cystectomy, Neoadjuvant chemotherapy

## Abstract

**Background:**

Neoadjuvant chemotherapy (NAC) with radical cystectomy (RC) is the preferred first-line treatment for localized muscle-invasive bladder cancer (MIBC). In recent years, octogenarians have been undergoing RC uneventfully, but studies showed older adults receive NAC less often. We studied the utilization and effect of RC with or without NAC in octogenarians and compared survival outcomes between responders and non-responders.

**Methods:**

In our retrospective study using the National Cancer Database (NCDB), we identified octogenarians with MIBC and urothelial histology who underwent RC with or without NAC between 2004 and 2018. The NAC cohort included patients who underwent RC with NAC, and the non-NAC cohort included those with or without adjuvant chemotherapy. The NAC cohort was subcategorized into responders and non-responders based on surgical pathology. Patients with comorbidity index > 1 were not included, thereby excluding patients with possible renal impairment. After propensity-matching, we compared the overall survival (OS) between NAC and non-NAC cohorts and responders and non-responders.

**Results:**

33924 patients underwent RC, and 3056 octogenarians met our selection. Among them, 396 received NAC, and 2660 did not receive NAC. Among those who received NAC, 112(28.3%) experienced downstaging, and 223(56.4%) exhibited upstaging or no change (*p* < 0.001). After propensity-matching, the median OS of the NAC and non-NAC cohorts were 51.6 months and 31.3 months, respectively (p<0.001). Similarly, the median OS of responders and non-responders were 89.4 months and 26.5 months, respectively(*p* < 0.0001).

**Conclusion:**

In our study, we observed that NAC with RC for MIBC may help to improve OS among healthy octogenarians. Similarly, responders had better OS than non-responders.

## Introduction

Bladder cancer is the 10th most common cancer worldwide and the 6th most common in the United States, with an estimated 82,290 new cases and 16,710 deaths attributed to it [1]. Bladder cancer is an aggressive disease associated with an increased risk of morbidity and mortality, and the median age at diagnosis is 73 years [2]. During diagnosis, approximately 20 to 30% of patients have muscle-invasive bladder cancer (MIBC) [3]. Currently, the average life expectancy for the US population is 76 years, and also, with the increase in life expectancy over decades and improvement in healthcare, many individuals in their eighties are undergoing major surgeries like radical cystectomy (RC) uneventfully [4].

Guidelines suggest neoadjuvant chemotherapy (NAC) with RC as the first-line treatment for localized MIBC, and its benefits were demonstrated in various randomized clinical trials [5, 6]. RC is highly effective for local control, and NAC deals with micrometastasis at diagnosis, tumor downstaging, and eliminating residual tumors [7]. Studies have shown platinum-based chemotherapy regimens with RC had a 5% increase in overall survival (OS) compared to locoregional therapy alone [8, 9]. Similarly, patients who received NAC had better 5-year OS than those who did not (60.6% vs. 49.1%) [10]. Studies in the elderly population have shown that NAC is underutilized [11]. Patients have better chances of completing NAC before RC as the tolerability may be challenged due to the morbidity following RC. To improve the oncological outcomes and quality of life in octogenarians undergoing RC, it is essential to understand the impact of NAC. This understanding can also facilitate informed and shared decision-making prior to RC. In this study, we use the National Cancer Database (NCDB) to analyze the trends in NAC utilization and compare the survival outcomes among octogenarians who underwent RC with NAC.

## Methods

### Study design

Our study is a retrospective study using NCDB, in which our initial cohort included patients aged 80 or more and localized MIBC (T2-4aN0M0) with urothelial histology between 2004 and 2018. The NCDB is a nationwide oncological database that compiles approximately 70% of all newly diagnosed cancers in the United States. The NCDB data used in the study is available as a de-identified dataset provided by the American Cancer Society upon request to the investigators of the participating institute. All information gathered is Health Insurance Portability and Accountability Act compliant. The data gathered in NCDB are periodically validated with quality monitoring on a yearly basis [12, 13]. Data entered in the NCDB are completely deidentified and not re-identifiable and don’t meet the definition of human subject research. Therefore, the Miami Cancer Institutional Review Board ethics approval and informed consent are not required. Survival outcomes between patients who received NAC and those who did not receive NAC were compared. Patients who met our selection criteria were divided into NAC and non-NAC cohorts based on neoadjuvant chemotherapy status. NAC cohort was defined as those who received multi-agent neoadjuvant chemotherapy within three months prior to radical cystectomy. The non-NAC cohort was defined as those who underwent only radical cystectomy and those who received adjuvant chemotherapy after radical cystectomy. Later, they were subcategorized into responders and non-responders based on the surgical pathology. Responders were defined as those with a decrease in the pathological stage to < = T2. Non-responders were those who had an increase or no change in the pathological stage. We excluded patients with non-urothelial histology, single-agent chemotherapy, Charlson-Deyo comorbidity index > 1 as it potentially excludes those with renal impairment who have comorbidity index 2, and missing data (Clinical T, Clinical N, treatment, follow-up, and vital status). Based on the inclusion and exclusion criteria, we identified 3056 octogenarians who were considered for our final analysis.

### Statistical analysis

Sociodemographic (sex, race, ethnicity, treatment facility, median income, and insurance) and clinical (comorbidity score, histology, tumor grade, and clinical stage) parameters were compared between the NAC and non-NAC cohorts. Race was simplified into Asian, Black, White, and unknown/others, and ethnicity was grouped as Hispanic or non-Hispanic. It was categorized so because fewer proportions of patients were found in those subgroups. Sociodemographic and clinical data were tabulated using Chi-square analysis and contingency tables. We utilized a matched propensity score methodology to mitigate the potential bias in the data. We developed a complex logistic regression model with or without NAC as the dependent variable. Our model included sex, race, ethnicity, comorbidity score, tumor grade, and clinical T stage. This method employed propensity scores with caliper widths < 0.25 times the standard deviation of the logit of the propensity score. It also used the nearest Mahalanobis measure to match each patient with (case) and without (control) NAC. Our matching algorithm systematically paired each NAC patient with the non-NAC patient whose propensity score fell within the specified caliper width. Patients with a compatible candidate match were included in the analysis. The equilibrium in variables was assessed before and after matching with standardized differences. An indication of balance was observed when the standardized difference was below 10%. A Kaplan-Meier curve and Cox regression analysis were used to compare the survival outcomes between NAC and non-NAC cohorts. Similarly, the overall survival was compared between the responders and non-responders. The statistical analysis was performed using IBM^®^ SPSS Statistics, version 28.0, and SAS.

## Results

Between 2004 and 2018, NCDB documented a total of 671,462 patients with bladder cancer. Among these patients, 95,543 had a clinical stage of T2-T4aN0M0. Within this cohort, 33,924 had undergone RC. Among those who underwent RC, 3056 octogenarians met our selection criteria. Among this octogenarian cohort, 396(12.9%) had received NAC, while 2660(87.1%) did not receive NAC. Subsequently, a propensity-matched cohort was generated, consisting of 396 patients in both the NAC and non-NAC groups.

**Fig. 1:**
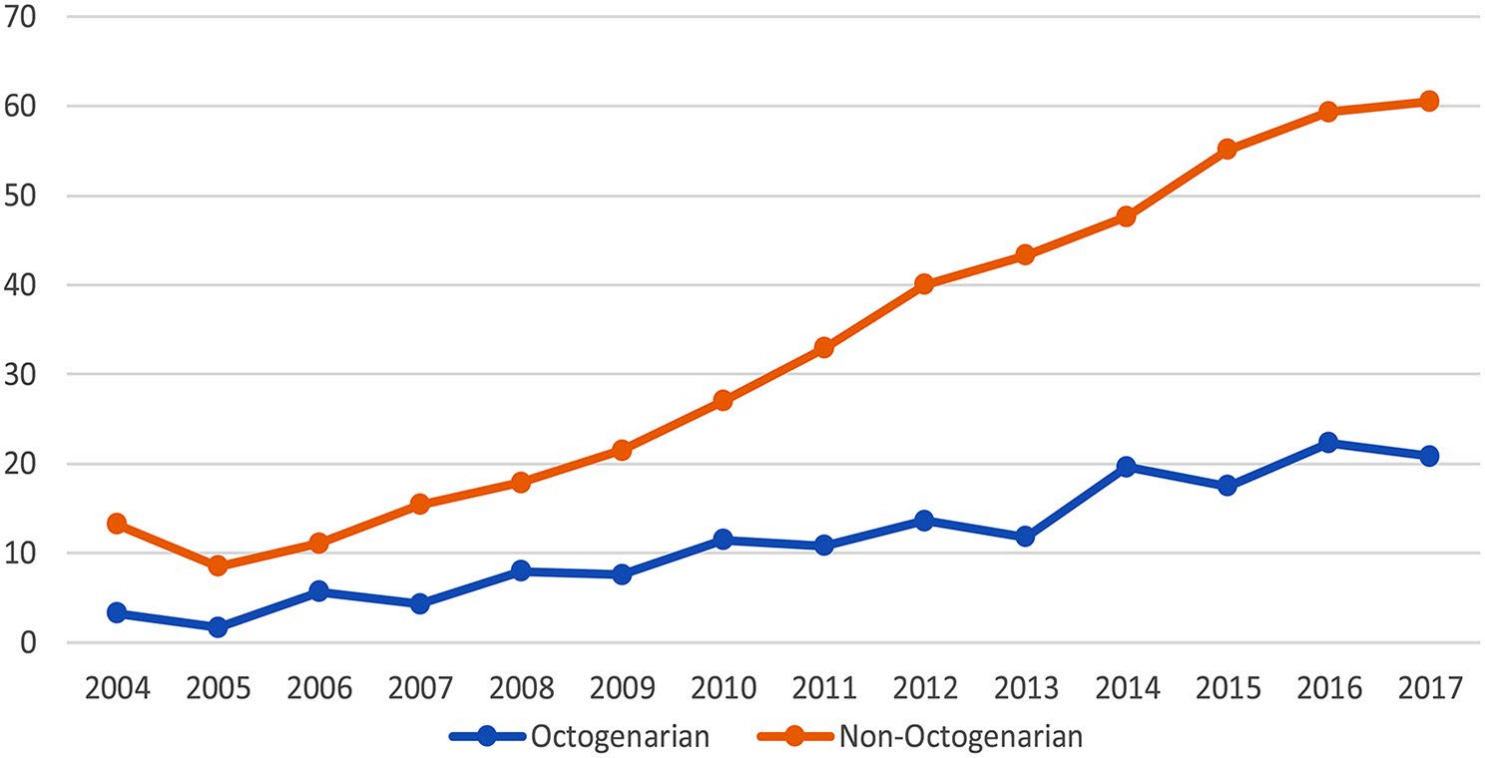
Utilization of NAC among Octogenarians and non-octogenarians. x-axis -Year of diagnosis; y-axis - % of NAC utilization

In the univariate analysis (Table 1), among the unmatched NAC cohort, a comorbidity score of 0 was seen in 315(79.5%) patients and a score of 1 in 81(20.5%). Similarly, among patients in the non-NAC cohort, 1,924(72.3%) had a comorbidity score of 0, and 736(27.7%) had a comorbidity score of 1. Pathological downstaging was seen in 112(28.3%) patients in the NAC cohort and 125(4.7%) in the non-NAC cohort. Pathological upstaging was seen in 142(35.9%) patients in the NAC cohort and 1223(46.0%) patients in the non-NAC cohort (*p* < 0.001). There was no change in pathological stage in 81(20.5%) patients in the NAC cohort and 816(30.7%) patients in the non-NAC (*p* < 0.001). Among the patients in NAC and non-NAC cohorts, 52(13.9%) and 31(1.2%) experienced a complete pathological response (pT0N0), respectively. There was no statistically significant difference in race, ethnicity, income, facility type, histology, surgical margin, and clinical stage. Adjuvant chemotherapy was used in 154(6.2%) patients in the non-NAC cohort. Utilization of neoadjuvant chemotherapy (Fig. 1) in octogenarians with MIBC has increased between 2004 and 2018. The utilization rate of NAC has been variable over the years, but it gradually increased from 3.3 to 20.8% among octogenarians and from 13.2 to 60.5% among non-octogenarians.

**Table 1:** Demographic parameters of the matched and unmatched population of NAC and non-NAC cohorts

Variable	Unmatched population		*p*-value	Matched population		*p*-value
	Non-NAC (*N* = 2660)	NAC (*N* = 396)		Non-NAC (*N* = 396)	NAC (*N* = 396)	
**Sex**			0.185			1.000
Male	1896 (71.3%)	295 (74.5%)		295 (74.5%)	295 (74.5%)	
Female	764 (28.7%)	101 (25.5%)		101 (25.5%)	101 (25.5%)	
**Race**			0.941			0.884
White	2518 (94.7%)	376 (94.9%)		381 (96.1%)	376 (94.9%)	
Black	76 (2.8%)	12 (3.0%)		8 (2.0%)	12 (3.0%)	
Asian	31 (1.2%)	3 (0.8%)		3 (0.8%)	3 (0.8%)	
Others/Unknown	35 (1.3%)	5 (1.3%)		4 (1.1%)	5 (1.3%)	
**Ethnicity**			0.400			0.723
Non-Hispanic	2493 (93.7%)	378 (95.5%)		376 (94.9%)	378 (95.5%)	
Hispanic	58 (2.2%)	6 (1.5%)		9 (2.3%)	6 (1.5%)	
Unknown	109 (4.1%)	12 (3.0%)		11 (2.8%)	12 (3.0%)	
**Facility Type**			0.826			0.996
Community CP	125 (4.7%)	17 (4.3%)		16 (4.0%)	17 (4.3%)	
Comprehensive CCP	782 (29.4%)	112 (28.3%)		110 (27.8%)	112 (28.3%)	
Academic Program	1331 (50%)	208 (52.5%)		210 (53.0%)	208 (52.5%)	
Integrated Network CP	422 (15.9%)	59 (14.9%)		60 (15.2%)	59 (14.9%)	
**Median Income**			0.133			0.164
<$40,227	347 (14.2%)	33 (9.8%)		55 (14.7%)	33 (9.8%)	
$40,277 − 50,353	553 (22.7%)	75 (22.2%)		85 (22.8%)	75 (22.2%)	
$50,354 − 63,332	604 (24.7%)	93 (27.5%)		103 (27.6%)	93 (27.5%)	
>=$63,333	937 (38.4%)	137 (40.5%)		130 (34.9%)	137 (40.5%)	
Missing	219	58		23	58	
**Insurance**			0.370			0.984
Not insured	10 (0.4%)	2 (0.5%)		3 (0.8%)	2 (0.5%)	
Private	200 (7.5%)	30 (7.6%)		29 (7.3%)	30 (7.6%)	
Medicaid	27 (1.0%)	9 (2.3%)		7 (1.8%)	9 (2.3%)	
Medicare	2380 (89.5%)	348(87.9%)		351 (88.6%)	348 (87.9%)	
Others	13(0.5%)	3(0.8%)		2 (0.5%)	3 (0.8%)	
Unknown	30 (1.1%)	4 (1.0%)		4 (1.0%)	4 (1.0%)	
**Comorbidity Index**			0.002			1.000
0	1924 (72.3%)	315 (79.5%)		315 (79.5%)	315 (79.5%)	
1	736 (27.7%)	81 (20.5%)		81 (20.5%)	81 (20.5%)	
**Clinical T**			0.840			0.899
cT2	2197 (82.6%)	331 (83.6%)		328 (82.8%)	331 (83.6%)	
cT3	323 (12.1%)	44 (11.1%)		48 (12.1%)	44 (11.1%)	
cT4	140 (5.3%)	21 (5.3%)		20 (5.1%)	21 (5.3%)	
**Pathological Response**			< 0.001			< 0.001
No Change	816 (30.7%)	81 (20.5%)		120 (30.3%)	81 (20.5%)	
Downstaging	125 (4.7%)	112 (28.3%)		22 (5.6%)	112 (28.3%)	
Upstaging	1223 (46.0%)	142 (35.9%)		173 (43.7%)	142 (35.9%)	
Unknown	496 (18.6%)	61 (15.4%)		81 (20.5%)	61 (15.4%)	
**Surgical Margin**			0.136			0.822
Negative	2194 (82.5%)	332(83.5%)		331 (83.6%)	332 (83.8%)	
Positive	345 (13%)	40 (10.1%)		44 (11.1%)	40 (10.1%)	
Unknown	121 (4.5%)	24 (6.1%)		21 (5.3%)	24 (6.1%)	

NAC – Neoadjuvant Chemotherapy; non-NAC – No Neoadjuvant chemotherapy; CP – Cancer Program; CCP – Community Cancer Program; cT – Clinical T stage

**Fig. 2:**
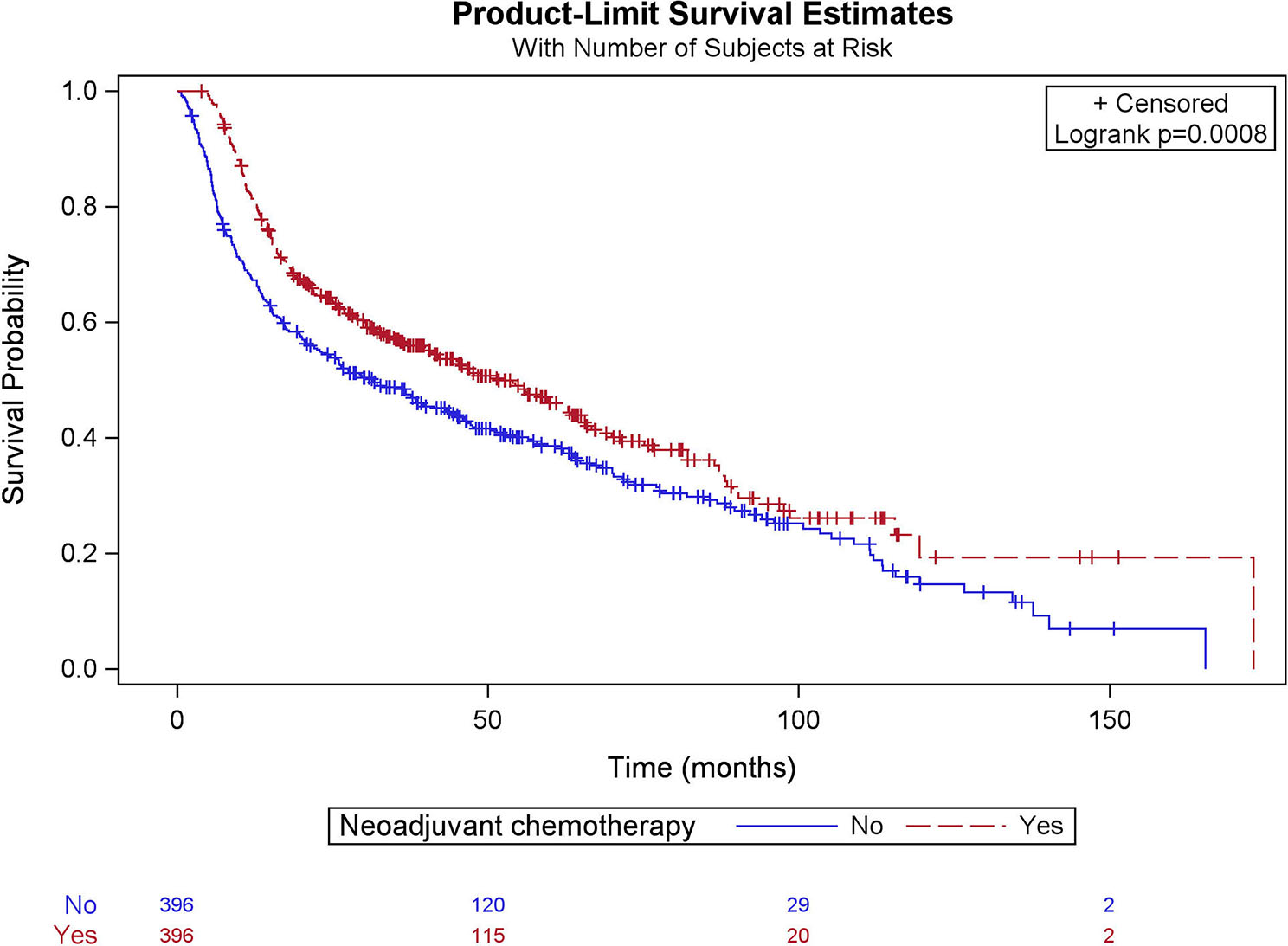
Comparison of overall survival between matched NAC and non-NAC cohorts among octogenarians

**Fig. 3:**
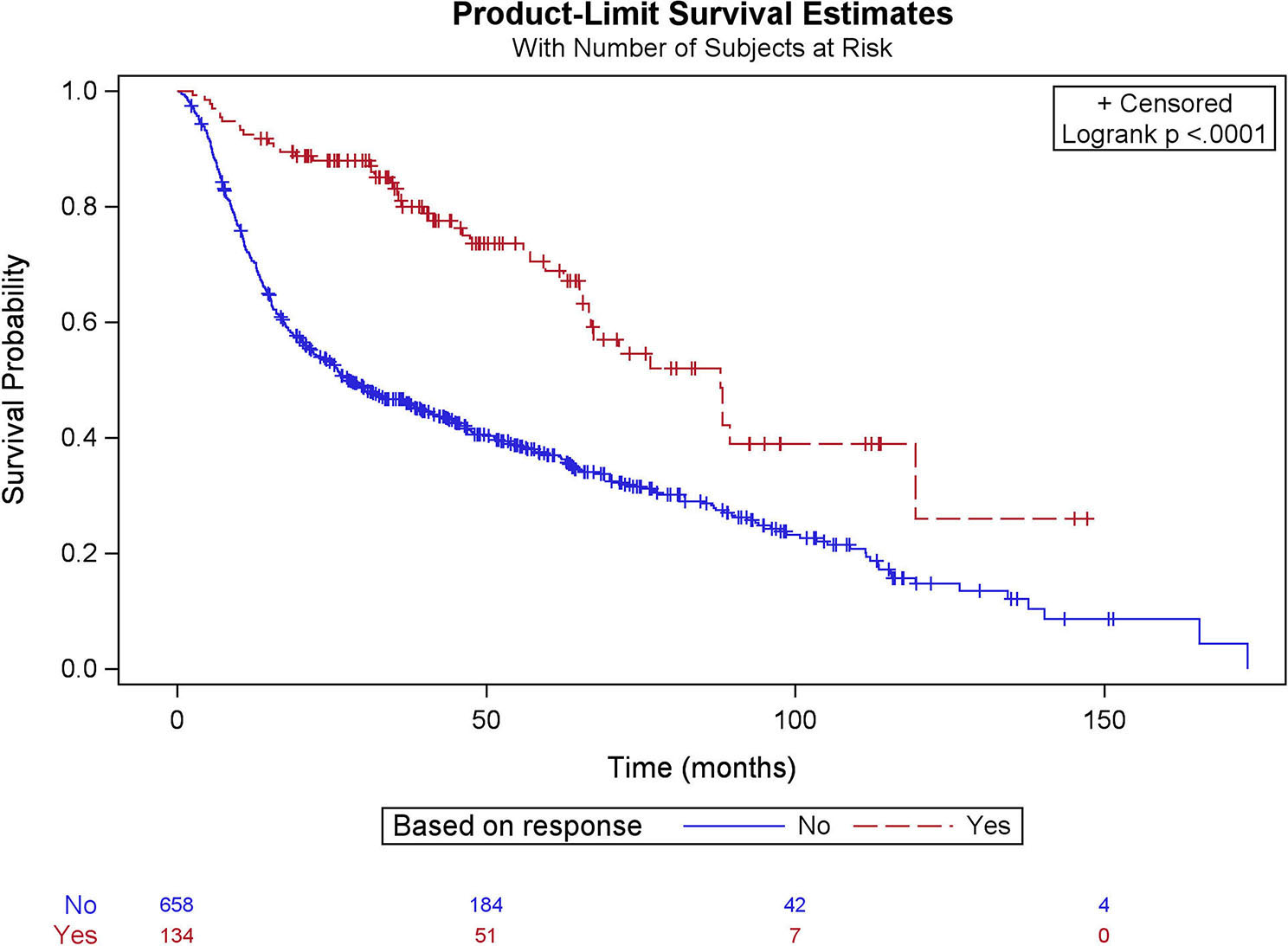
Comparison of overall survival between matched responders and non-responders among octogenarians

In the multivariate Cox regression analysis (Table 2), within the NAC cohort, patients with pathological downstaging had reduced mortality risk, with a Hazards ratio (HR) of 0.57 (*p* < 0.005), and patients with pathological upstaging had an increased mortality risk with HR = 1.89 (*p* < 0.001). Similarly, patients with positive surgical margins had an increased risk of mortality with HR = 1.91 (*p* < 0.0001). Kaplan- Meier survival analysis comparing the matched NAC and non-NAC cohorts showed (Fig. 2) that the OS for the NAC cohort was 51.6 (95% CI; 40.1–63.2) months, and the non-NAC cohort was 31.3 (95% CI; 22.9–43.3) months (*p* < 0.001). Similarly, OS for the matched responders and non-responders (Fig. 3) was 89.4 (95% CI; 76.6-119.4) months and 26.5 (95% CI; 25.0–29.0) months, respectively (*p* < 0.001).

**Table 2:** Adjusted Cox regression model for association between NAC and overall survival

Parameter	Hazard ratio	*p*-value
**Sex**		
Male	Reference	
Female	0.86 (0.68–1.08)	0.1823
**Race**		
White	Reference	
Black	1.35 (0.71–2.56)	0.3616
Asian	0.89 (0.30–2.67)	0.8400
Others	2.95 (0.56–15.65)	0.2032
**Ethnicity**		
Non-Hispanic	Reference	
Hispanic	0.38 (0.16–0.91)	0.0296
**Facility Type**		
Community CP	Reference	
Comprehensive CCP	0.81 (0.50–1.30)	0.3786
Academic Program	0.87 (0.55–1.38)	0.5525
Integrated Network CP	0.96 (0.58–1.58)	0.8594
**Comorbidity Index**		
0	Reference	
1	1.29 (1.02–1.63)	0.0355
**Clinical T**		
cT2	Reference	
cT3	0.98 (0.72–1.34)	0.9057
cT4	1.58 (1.04–2.41)	0.0332
**Pathological Response**		
No Change	Reference	
Downstaging	0.57 (0.39–0.84)	0.0048
Upstaging	1.89 (1.47–2.43)	< 0.0001
Unknown	1.36 (1.01–1.82)	0.0400
**Neoadjuvant Chemotherapy**		
No NAC	Reference	
Yes NAC	0.88 (0.73–1.08)	0.2230
**Surgical Margin**		
Negative	Reference	
Positive	1.91 (1.44–2.54)	< 0.0001
Unknown	0.82 (0.54–1.24)	0.3476

CP – Cancer Program; CCP – Community Cancer Program; cT – Clinical T stage; NAC – Neoadjuvant Chemotherapy

## Discussion

Our study on NAC in octogenarians comparing the survival outcomes between the NAC and non-NAC showed that the utilization of NAC has gradually increased over the years but is still relatively less compared to the younger counterparts. Approximately one-third of the patients who received NAC experienced downstaging. Patients who received NAC had better overall survival compared to those who did not receive NAC (51.6 vs. 31.3 months). Similarly, the responders had a reduced risk of mortality and better overall survival compared to non-responders (89.4 vs. 26.5 months). Therefore, NAC could be beneficial in carefully selected individuals in this subset of patients.

A study by McFerrin et al. from NCDB has shown that the trends in perioperative chemotherapy utilization have gradually increased from 2011 to 2015 (46.4–57.5%). NAC increased from 22.9 to 32.3%, and adjuvant chemotherapy had no significant change [14]. Studies from the SEER database have shown that from 2004 to 2013, the odds of receiving NAC increased by 27.5%, while the odds of receiving AC decreased by 7.5%. Similarly, the use of NAC in patients with T2 tumors increased by 15.9 to 66.2%. They also observed that the female gender, lower comorbidity index, and lower-stage disease had increased odds of receiving NAC [15, 16]. In our study, we observed that the utilization of NAC has increased variably over the years in octogenarians between 2004 and 2018 from 3.3 to 20.8%, which was proportionally less compared to younger populations (13.2–60.5%). This difference in chemotherapy utilization among older adults may be due to concerns about tolerability.

Studies have shown that the NAC group had better 5-year OS than the non-NAC group, and the NAC group had higher recurrence-free survival and cancer-specific survival than the non-NAC group [17, 18]. Similarly, the NAC group also had better pathological responses, and those with pathological downstaging had better OS benefits than those who did not receive NAC [19, 20]. A study by Russell et al. from Bladder Cancer Database Sweden showed no significant difference in 5-year survival but a decreased risk of overall and cancer-specific death in the NAC group [21]. Our study also observed that the NAC cohort had better OS than the non-NAC cohort, and the responders had better OS than non-responders. In a study by Soria et al. on cT2N0M0 patients, the NAC group had better complete (30% vs. 3%) response and downstaging (55% vs. 24%) compared to the non-NAC group but no significant difference in OS [22]. Studies have shown patients with positive surgical margins after RC have poor survival outcomes, and adjuvant therapies can be adapted based on surgical margin being considered as a predictor [23, 24]. In our study, there was no significant difference in positive surgical between NAC and non-NAC groups, but there was increased mortality risk in patients with positive surgical margins and only limited data on adjuvant therapies. We also noted that the NAC cohort had a better complete response (13.9% vs. 1.2%) and downstaging (28.3% vs. 4.7%) than the non-NAC cohort. This difference in the non-NAC cohort could have been due to limited transurethral resection, considering the extent of the disease during diagnosis.

There have been concerns about NAC tolerability and its impact on survival outcomes in the elderly population, which has been explored in various studies. Leone et al. reported that although glomerular filtration rate, performance status, and pre-operative hemoglobin were less in patients 70 years and above than their younger counterparts, there was no significant difference in recurrence-free survival and overall survival [25]. Similarly, Chau et al. showed cisplatin-based NAC in patients above 70 and younger populations had similar 3-year OS, and the pathological response was 31.3% and 40%, respectively [26]. A study by Posielski et al. observed 30-day readmission, 30- and 90-day mortality, and OS was better with NAC compared to those who did not receive NAC [11]. These studies show the tolerability and the outcomes following NAC in the older population are considerate. Our analysis also showed that the patients who received NAC had better pathological response and OS than those who did not receive NAC, and the responders had better OS than the non-responders. Studies have been conducted to identify genomic biomarkers that could assess the response to platinum-based NAC. Positive association with ERCC2, ATM, RB1, and FNACC gene variations and pathological and survival outcomes has been demonstrated, and also the relationship between PD-L1-expressing tumors and neoadjuvant immunotherapy has been studied [27, 28]. Using platinum-based chemotherapy regimens in older adults has always been a concern as renal function deteriorates with age. Studies on genomic biomarkers to identify the responders could help in the effective utilization of NAC.

Over decades, with the increase in life expectancy and improvement in health care, individuals in their eighties undergo major surgeries. The average life expectancy of an octogenarian ranges between 8 and 10 years depending on the associated comorbidities; hence, the benefits of NAC could help to improve survival outcomes among carefully selected individuals in this age group. Stratifying patients based on performance status, fragility index, and other measures could help us carefully select candidates among octogenarians for NAC. Our study has several limitations. First, it is a retrospective study subjected to various biases, like sampling bias, as NCDB records only the data from CoC-accredited facilities. Though propensity matching was performed, the bias cannot be eliminated completely. Details on the chemotherapy regimen, dosage, number of cycles, and related complications or toxicity were not available. We also lack survival data like cancer-specific, recurrence-free, and metastasis-free survival. These would be useful for assessing the efficacy of various chemotherapy regimens. There were limited patients in this subset of the population with variant histology. Hence, further studies are required to understand the impact of NAC in variant histology. Even though this is a database-related study, results warrant careful interpretation. Also, information on performance status, frailty index, and quality of life following chemotherapy was not available and could be valuable for planning personalized treatment strategies for elderly individuals.

## Conclusion

In our study on octogenarians, we observed that despite the underutilization of NAC, approximately one-fourth of patients who received NAC experienced pathological downstaging. Patients who received NAC had better OS than those who did not receive NAC, and the responders had better OS than the non-responders. This study helps us understand the impact of NAC with radical cystectomy on carefully selected octogenarians, which could help us with patient counseling and shared-decision making. Prospective studies on the predictive genomic biomarkers and their influence on NAC will provide valuable insight in selecting suitable candidates among octogenarians for NAC and in designing an optimal regimen.

## Data Availability

The data used in the study is available at the National Cancer Database Repository. http://www.facs.org/quality-programs/cancer/ncdb.

## Abbreviations

NCDB: National Cancer Database
NAC: Neoadjuvant Chemotherapy
Non-NAC: No Neoadjuvant Chemotherapy
RC: Radical Cystectomy
OS: Overall Survival

## References

[1] Siegel RL, Miller KD, Wagle NS, Jemal A. Cancer statistics, 2023. CA Cancer J Clin. 2023;73(1):17–48. 36633525 10.3322/caac.21763

[2] Fang W, Yang ZY, Chen TY, Shen XF, Zhang C. Ethnicity and survival in bladder cancer: a population-based study based on the SEER database. J Transl Med. 2020;18(1):145. 32228610 10.1186/s12967-020-02308-w PMC7106682

[3] Hanna N, Trinh QD, Seisen T, Vetterlein MW, Sammon J, Preston MA, et al. Effectiveness of Neoadjuvant Chemotherapy for muscle-invasive bladder Cancer in the current Real World setting in the USA. Eur Urol Oncol. 2018;1(1):83–90. 31100232 10.1016/j.euo.2018.03.001

[4] Xu J, Murphy SL, Kochanek KD, Arias E. Mortality in the United States, 2021. NCHS Data Brief. 2022(456):1–8. 36598387

[5] Grossman HB, Natale RB, Tangen CM, Speights VO, Vogelzang NJ, Trump DL, et al. Neoadjuvant chemotherapy plus cystectomy compared with cystectomy alone for locally advanced bladder cancer. N Engl J Med. 2003;349(9):859–66. 12944571 10.1056/NEJMoa022148

[6] Witjes JA, Bruins HM, Cathomas R, Compérat EM, Cowan NC, Gakis G, et al. European Association of Urology Guidelines on muscle-invasive and metastatic bladder Cancer: Summary of the 2020 guidelines. Eur Urol. 2021;79(1):82–104. 32360052 10.1016/j.eururo.2020.03.055

[7] Hamid A, Ridwan FR, Parikesit D, Widia F, Mochtar CA, Umbas R. Meta-analysis of neoadjuvant chemotherapy compared to radical cystectomy alone in improving overall survival of muscle-invasive bladder cancer patients. BMC Urol. 2020;20(1):158. 33054762 10.1186/s12894-020-00733-z PMC7557048

[8] Yin M, Joshi M, Meijer RP, Glantz M, Holder S, Harvey HA, et al. Neoadjuvant chemotherapy for muscle-invasive bladder Cancer: a systematic review and two-step Meta-analysis. Oncologist. 2016;21(6):708–15. 27053504 10.1634/theoncologist.2015-0440 PMC4912364

[9] Griffiths G, Hall R, Sylvester R, Raghavan D, Parmar MK. International phase III trial assessing neoadjuvant cisplatin, methotrexate, and vinblastine chemotherapy for muscle-invasive bladder cancer: long-term results of the BA06 30894 trial. J Clin Oncol. 2011;29(16):2171–7. 21502557 10.1200/JCO.2010.32.3139 PMC3107740

[10] Fahmy O, Khairul-Asri MG, Schubert T, Renninger M, Malek R, Kübler H, et al. A systematic review and meta-analysis on the oncological long-term outcomes after trimodality therapy and radical cystectomy with or without neoadjuvant chemotherapy for muscle-invasive bladder cancer. Urol Oncol. 2018;36(2):43–53. 29102254 10.1016/j.urolonc.2017.10.002

[11] Posielski N, Koenig H, Ho O, Porter C, Flores JP. Use of Neoadjuvant Chemotherapy in Elderly patients with muscle-invasive bladder Cancer: a Population-based study, 2006–2017. Oncol (Williston Park). 2022;36(1):21–33. 10.46883/2022.25920939 35089670

[12] Bilimoria KY, Stewart AK, Winchester DP, Ko CY. The National Cancer Data Base: a powerful initiative to improve cancer care in the United States. Ann Surg Oncol. 2008;15(3):683–90. 18183467 10.1245/s10434-007-9747-3 PMC2234447

[13] Boffa DJ, Rosen JE, Mallin K, Loomis A, Gay G, Palis B, et al. Using the National Cancer Database for Outcomes Research: a review. JAMA Oncol. 2017;3(12):1722–8. 28241198 10.1001/jamaoncol.2016.6905

[14] McFerrin C, Davaro F, May A, Raza S, Siddiqui S, Hamilton Z. Trends in utilization of neoadjuvant and adjuvant chemotherapy for muscle invasive bladder cancer. Investig Clin Urol. 2020;61(6):565–72. 32985142 10.4111/icu.20200132 PMC7606117

[15] Macleod LC, Yabes JG, Yu M, Fam MM, Hale NE, Turner RM 2, et al. Trends and appropriateness of perioperative chemotherapy for muscle-invasive bladder cancer. Urol Oncol. 2019;37(7):462–9. 10.1016/j.urolonc.2019.04.006 31053530

[16] Mazzone E, Knipper S, Mistretta FA, Tian Z, Preisser F, Gallina A, et al. Is neoadjuvant chemotherapy for pT2 bladder cancer associated with a survival benefit in a population-based analysis? Cancer Epidemiol. 2019;58:83–8. 30528834 10.1016/j.canep.2018.11.007

[17] Nitta M, Kuroda S, Nagao K, Higure T, Zakoji H, Miyakita H, et al. Effect of neoadjuvant chemotherapy in patients undergoing radical cystectomy for muscle-invasive bladder cancer: a retrospective, multi-institutional study. Jpn J Clin Oncol. 2020;50(1):73–9. 31612911 10.1093/jjco/hyz137

[18] Kubota M, Kanno T, Inoue T, Yamasaki T, Okumura K, Ito K, et al. Effect of optimal neoadjuvant chemotherapy on oncological outcomes of locally advanced bladder cancer with laparoscopic radical cystectomy: a matched-pair analysis in a multicenter cohort. Int J Urol. 2021;28(6):656–64. 33682243 10.1111/iju.14533

[19] Møller CT, Støer NC, Blindheim A, Berge V, Tafjord G, Fosså SD, et al. Downstaging and survival after neoadjuvant chemotherapy for bladder cancer in Norway; a population-based study. BMC Cancer. 2022;22(1):1301. 36510166 10.1186/s12885-022-10394-w PMC9746207

[20] van Ginkel N, Hermans TJN, Meijer D, Boormans JL, Voortman J, Mertens L, et al. Survival outcomes of patients with muscle-invasive bladder cancer according to pathological response at radical cystectomy with or without neo-adjuvant chemotherapy: a case-control matching study. Int Urol Nephrol. 2022;54(12):3145–52. 35997906 10.1007/s11255-022-03339-6 PMC9606088

[21] Russell B, Sherif A, Häggström C, Josephs D, Kumar P, Malmström PU, et al. Neoadjuvant chemotherapy for muscle invasive bladder cancer: a nationwide investigation on survival. Scand J Urol. 2019;53(4):206–12. 31174452 10.1080/21681805.2019.1624611

[22] Soria F, Black PC, Fairey AS, Cookson MS, Yu EY, Kassouf W, et al. Neoadjuvant chemotherapy plus radical cystectomy versus radical cystectomy alone in clinical T2 bladder cancer without hydronephrosis. BJU Int. 2021;128(1):79–87. 33152179 10.1111/bju.15289

[23] Claps F, van de Kamp MW, Mayr R, Bostrom PJ, Boormans JL, Eckstein M, et al. Risk factors associated with positive surgical margins’ location at radical cystectomy and their impact on bladder cancer survival. World J Urol. 2021;39(12):4363–71. 34196758 10.1007/s00345-021-03776-5

[24] Marcq G, Afferi L, Neuzillet Y, Nykopp T, Voskuilen CS, Furrer MA et al. Oncological outcomes for patients harboring positive Surgical margins following radical cystectomy for muscle-invasive bladder Cancer: a Retrospective Multicentric Study on Behalf of the YAU Urothelial Group. Cancers (Basel). 2022;14(23). 10.3390/cancers14235740 PMC9739538 36497222

[25] Leone AR, Zargar-Shoshtari K, Diorio GJ, Sharma P, Boulware D, Gilbert SM, et al. Neoadjuvant Chemotherapy in Elderly patients with bladder Cancer: oncologic outcomes from a single Institution experience. Clin Genitourin Cancer. 2017;15(4):e583–9. 28410909 10.1016/j.clgc.2017.01.014 PMC7771283

[26] Chau C, Wheater M, Geldart T, Crabb SJ. Clinical outcomes following neoadjuvant cisplatin-based chemotherapy for bladder cancer in elderly compared with younger patients. Eur J Cancer Care (Engl). 2015;24(2):155–62. 25620269 10.1111/ecc.12282

[27] Gil-Jimenez A, van Dorp J, Contreras-Sanz A, van der Vos K, Vis DJ, Braaf L, et al. Assessment of Predictive genomic biomarkers for response to cisplatin-based Neoadjuvant Chemotherapy in bladder Cancer. Eur Urol. 2023;83(4):313–7. 35965206 10.1016/j.eururo.2022.07.023

[28] Necchi A, Raggi D, Gallina A, Madison R, Colecchia M, Lucianò R, et al. Updated results of PURE-01 with preliminary activity of Neoadjuvant Pembrolizumab in patients with muscle-invasive bladder carcinoma with variant histologies. Eur Urol. 2020;77(4):439–46. 31708296 10.1016/j.eururo.2019.10.026

